# Hyped-up or meditate: A scoping review of mindfulness-based group interventions for adolescents with attention deficit hyperactivity disorder

**DOI:** 10.1177/13591045241272835

**Published:** 2024-08-08

**Authors:** Lucy Tan, Maria Jones

**Affiliations:** 1 Clinical Psychology, 8001 James Cook University, Singapore; 2 Department of Health Psychology, 8863 RCSI University of Medicine and Health Sciences, Ireland

**Keywords:** Adolescents, attention deficit hyperactivity disorder, hyperactivity, impulsivity, group mindfulness-based interventions, mental health

## Abstract

The objective of this scoping review is to evaluate the effectiveness of mindfulness training in improving functioning in adolescents (aged 12–19 years) diagnosed with Attention Deficit Hyperactivity Disorder (ADHD). Previous research has demonstrated that psychological interventions improve functioning in a myriad of domains for individuals diagnosed with ADHD, such as attention training, interpersonal relationships, and social skills. Mindfulness-based interventions (MBI) are indicated as an intervention in attention training. It maybe argued that group based MBI programmes should begin early, for children and adolescents at a time that is critical in their development. Methods and reporting are in line with the PRISMA extension for scoping reviews, the protocol is preregistered in the (Open Science Framework register). The study outcomes included attention, impulsivity, and relationships of adolescents with ADHD. Findings demonstrated preliminary evidence for the use of group-based mindfulness interventions with adolescents continues to be nascent. Although studies reported positive results, the evidence of its effectiveness for adolescents with ADHD is inconclusive, due to limited studies available and the limitations of the study design. This scoping review provides a panorama of MBI for ADHD adolescents.

## Plain language summary

This review explores group mindfulness-based programmes for adolescents with attention deficit hyperactive disorders. It provides a useful summary for clinicians.

## Introduction

The prevalence of Attention Deficit Hyperactivity Disorder (ADHD) in childhood and adolescence is one of the most common neurodivergent presentations in paediatrics and psychiatry in Western countries (Polanczyk et al., 2007). This is also similar in Asia, where ADHD was noted to be the leading diagnosis at the Singapore Institute of Mental Health clinics (Lim et al., 2015). ADHD is defined as a persistent pattern of hyperactivity, impulsivity and inattention which is higher than that expected for the individual’s developmental level (Diagnostic and Statistical Manual of Mental Disorders, Fifth Edition (DSM-5), American Psychiatric Association, 2013). Symptoms must be present for at least six months in more than two settings, have appeared before the age of 12 years, and they must interfere with the normal functioning of the individual. The three subtypes of ADHD are: inattentive; hyperactive-impulsive and combined subtypes.

This syndrome results in a deficit in the executive control of behaviour, with negative outcomes manifesting during both childhood and adulthood and impacting on multiple domains of the individual’s life such as academic, family, and social development (Barkley, 1997; see Barkley, 2006 for a review). Beyond the individual, the impact of ADHD is also a considerable burden at a familial system and societal level (Coghill & Hodgkins, 2016; Mulraney & Coghill, 2018).

The 1990–2019 Global Burden of Disease (GBD 2019) study reported the largest increase in incidence, prevalence and burden observed in the USA.; further analyses demonstrated incidence peaked at ages 5–9 years; and disability-adjusted life-years (DALYS) at age 10–14 years. Similarly, studies elsewhere estimated a worldwide pooled prevalence of around 5.3% in children and adolescents (Polanczyk et al., 2007); with ADHD symptoms persisted into adulthood in 70% of the individuals with a childhood diagnosis. The GBD study also found higher ADHD-related mortality attributable to unnatural causes.

In an Australian study (Economics DA, 2019) with a population of 24.6 million, ADHD is estimated to cost AUD$20.57 billion (GBP£11.3 billion), which translates to AUD$836 (GBP£459) per capita. Of this, total 63% were attributable to financial costs and 37% to well-being costs (i.e., costs associated with reduced quality of life and impaired functioning, and premature death). Similarly, in the U.S. it is estimated more than US$50 billion annually - akin to the societal cost of major depression and stroke (Pelham et al., 2007); demonstrating ADHD is a major public health concern.

The National Institute for Health and Care Excellence (NICE) ADHD guidelines (NICE, 2018) identified the need for information targeting various groups, with the objectives of better understanding symptoms, reducing stigma and prejudice, promoting understanding, better treatment, and support in settings such as education, physical health care and employment and increasing self-understanding. There is an opportunity to provide positive information, which can mitigate stress experienced by families and individuals with ADHD and reduce stigma associated with the condition.

Furthermore, international guidelines for treatment of ADHD suggest that methylphenidate for children with ADHD is, to date, still the first-line treatment (Walkup, 2009). In terms of cost effectiveness of medication versus behavioural treatment, medication seems to be the preferred option - the estimated medical costs per child with ADHD is $1079 compared to behavioural treatment per child is $7176 during the same 14 months period (Jensen et al., 2005). However, concerns about the frequency of methylphenidate prescriptions and its disadvantages are increasing (Graham et al., 2011; International Narcotics Control Annual Report, 2015). The stimulant medication use is not without side effects, e.g., insomnia, loss of appetite, abdominal pain, headache, anxiety, stress, and nervousness (Charach et al., 2004; Graham et al., 2011; Schachter et al., 2001; Storebo et al., 2015; Taylor et al., 2004). The National Institute of Mental Health (NIMH) Collaborative Multisite Multimodal Treatment Study of Children with ADHD Study (Connors et al., 2001) reported 64.1 % of the children suffered from one or more mild to severe side effects. Stimulant medication works only short-term, and symptoms return once medication is stopped (Abikoff et al., 2004; Brown et al., 1986; Taylor et al., 2004). Therefore, there is need to examine alternative treatments for adolescent ADHD.

### Rationale of mindfulness-based interventions and ADHD

Mindfulness training is based on over 2500-year history in Eastern traditions and philosophy; originally grounded in Buddhism. However, it is a relatively recent construct and practice in Western psychology (Brown et al., 2007). Mindfulness is frequently described as self-regulation of attention to the present moment experiences, approached and maintained with a non-judgmental stance, openness, and acceptance (Hayes & Fieldman, 2004; Tan & Martin, 2012).

Mindfulness practice involves focused attention, described by an open attitude, monitoring of attention with an initial focus on breath that builds concentrated attention; and with a broadening of the focused attention towards a steady monitoring of ‘whatever arises’ from sensory, mental, or emotional states (Kabat-Zinn, 1990). The ability to control and regulate attention to one’s reactivity to stress and negative valence, enhances self-regulation resulting in psychological well-being (Chambers et al., 2008). The clinical impact of mindfulness-based interventions such as Mindfulness-Based Cognitive Therapy (MBCT), Mindfulness-Based Stress Reduction (MBSR) and Taming the Adolescent Mind (TAM) in mental health conditions, e.g., depression, anxiety, and pain management, have been established and published elsewhere (for reviews refer to Mitchell, et al., 2015; Shapiro & Schwartz, 2000; Tan, 2015). From neuroimaging studies, neuroplasticity changes in attentional functioning have also been observed after mindfulness meditation (Mitchell et al., 2015). Further studies indicated that mindfulness training may improve attentional networks (Jha et al., 2007), increase cortical thickness (Lazar et al., 2005) and alter dopamine levels (Kjaer et al., 2002). The mind-body connection practices such as yoga, meditation and mindfulness can change brain activation patterns. As such, its potential benefits warrant further exploration on symptoms of ADHD in adolescents. Although the effects of mindfulness training in adults are well established, research on the effectiveness and efficacy of mindfulness training in child and adolescent psychiatry is less robust; with most of the research conducted with children and adolescents in non-clinical samples (Tan, 2015; Zoogman et al., 2014). The ability to anchor and sustain attention in the present moment whenever it wandered off, may be especially helpful for adolescents diagnosed with ADHD – as inattention is one of the core symptoms of ADHD. By observing, noticing, and accepting of ongoing streams of internal and external stimuli that enter one’s awareness, consequentially to choose how to respond, rather than to react on automatic pilot. Hence, targeting the other core symptoms of ADHD hyperactivity and impulsivity.

Additionally, the growing interest of Mindfulness-Based Interventions (MBIs) have been increasingly used in combination with conventional treatments in mental health. MBIs could play a role as augmentation strategies in ADHD since they have shown they can improve attention, awareness, sense of self and executive functions, and reduce impulsivity, emotional dysregulation, and stress levels (Baijal and Gupta, 2008; Hylander et al., 2017; Kozasa et al., 2012; Krisanaprakornkit et al., 2010; Lutz et al., 2008; Rapport et al., 2002; Schonert-Reichl et al., 2015; Tsai and Chou, 2016).

The extant systematic reviews of studies on MBIs and ADHD have been promising, albeit still not definitive, conclusions. Overall, existing reviews have mainly focused on the effectiveness of mindfulness (or related interventions, such as meditation and yoga) in improving ADHD symptoms. The evidence of MBIs efficacy among adult patients seems to be stronger than that detected in children.

This review aims to explore emergent group MBI for adolescents with ADHD (age 12–19 years old) providing a panoramic review and evaluate the efficacy of group MBIs in improving ADHD symptoms. We have focused on group MBIs, as the need to “hang out” in groups are a natural setting for young people (Erikson, 1968). In social and applied contexts, group work in the public health is a useful clinical practice for adolescents with varying mental health issues (Cramer-Azima, 2002). This review examines what MBI group-based programmes are utilised and what are the research and knowledge gaps?

## Method

### Literature search strategy

The research questions were informed by an initial literature search and tested search terms for suitability. Initial scan identified a lack of studies in group-based mindfulness training in adolescents with ADHD. A scoping review would be most suitable to provide a broad overview. The protocol for this review was developed using Arksey and O’Malley (2005) framework, with enhancements from Levac et al. (2010) to assess the methodological quality of the included studies. Methods and reporting are in line with the PRISMA extension for scoping reviews (Tricco et al., 2018); with its protocol preregistered in Open Science Framework depository.

The CINAHL, Cochrane, PubMed, PsycINFO and EBsco databases were searched for studies published from 2015 to 2023. The cut-off date of 2015 was selected because previous reviews on mindfulness interventions with children and adolescent were published (Burke, 2010; Tan, 2015). The search terms and parameters were: (1) adolescents diagnosed with ADHD (e.g., “hyperactive”, “inattentive”); (2) keywords used included ‘ADHD’, ‘youth’, ‘adolescent’, ‘group treatment’, ‘mindfulness intervention’ and ‘group programmes’; (3) only studies published in English were included. Conference papers and unpublished dissertations were excluded. Studies were identified by title and abstract to assess whether they met the inclusion criteria, reference list of relevant studies were also included in the search strategy. The risk of bias was minimised using the PRISMA checklist (Tricco et al., 2018) and studies reviewed independently by both authors.

### Inclusion and exclusion criteria

The inclusion criteria for studies in the review were as follows:

(1) For between-groups designs, at least one treatment group must utilise and describe group-based mindfulness intervention or practices (with or without home practices outside intervention sessions); with the focus to improve the main ADHD symptoms in adolescents (12 – 19 years old),
(2) Adolescents with ADHD including subtypes - inattentive, impulsive, or combined type,
(3) Studies providing quantitative data and inclusions of parallel parent training group MBI.

The exclusion criteria were:

(1) MBI as a minor subcomponent, combined with psychoeducation group programmes as the focus; making it difficult to evaluate the effectiveness of mindfulness on ADHD core symptoms,
(2) Single trials, case studies, individual treatment intervention, i.e., non-group based
(3) Experimental or laboratory studies.

### Data collection

The first author conducted the search, next both authors independently, conducted standardized assessments to determine study eligibility, according to the inclusion and exclusion criteria. The titles and abstracts were screened, and then full text retrieved; both authors analysed the full text for all the records deemed relevant. There were no disagreements and consensus were reached.

The first author extracted and tabulated the data using Microsoft Excel. A table was created, details of the studies were reviewed and organized according to several themes which were determined using the research aims and questions identified earlier (see Table 1 for a summary); to identify research gaps and appraisals of the methodologies, findings, and recommendations for future research in each paper – these are incorporated in the Discussion section.

**Table 1.** Summary of studies reviewed.

Study	Country	Sample	Age (years)	Intervention	Study design	Randomize	Control	Measures	Intervention findings/outcomes
Abdolahzadeh et al. (2017)	Iran	*N* = 30 females	13–18	8 weeks, 90 min/session with home practice, based on original MBSR programme	Pre-post	Yes	Yes	SNAP-IV, MAAS	Decrease in ADHD symptoms; increase in mindfulness
Haydicky et al. (2015)	Canada	*N* = 18 *N* = 17 parents	13–18	MYMind (a MBCT adaptation); 8 weeks, 1.5 hour/session with home practice and separate 8 weeks parallel course for parents	4 weeks prior intervention (baseline/control), pre-post & F/up	No	Yes	WASI, connors 3 – Parent & adolescent self-report, RCADS, SIPA, FAD, issues checklist, AAQ, IM-P	No significant improvement in adolescents’ attention; parents reported improvements in adolescents
Kiani et al. (2017)	Iran	*N* = 30 females	13–15	8 weeks, 90 min/session mi training, adapted from mindfulness prescription for adult ADHD, home practice	Pre-post, 1 month F/up	Yes	Yes	SNAP-IV, DERS, CPT, stroop, tower of London	Executive function in laboratory tasks planning and inhibition higher in treatment group
Roux et al. (2021)	Belgium	*N* = 129 adolescents in residential home	12–19	Two-part programme comprised of 6 weeks, 50 min/session psychoeducation followed by 10 weeks, 50 min/session mi practice	Pre-post	Yes	Yes, TAU & health psychoeducation	CBCL. MDI-C, CAMM, SNAP-IV, focused attention test-revised, waiting task, impulsive behavior scale, cookies task, lottery task	No significant improvement on mindfulness, impulsivity, hyperactivity compared to control conditions
Roux & Philippot (2020)	Belgium	*N* = 48 adolescent males in residential home	12–19	Two-part programme comprised of 6 weeks, 50 min/session psychoeducation followed by 10 weeks, 50 min/session mi practice	Pre-post, quasi-experimental	Yes	Yes, wait-list	CBCL, MDI-C, SNAP-IV, impulsive behavior scale	Decreased in impulsivity and externalizing symptoms in MI group; both decreased depressive symptoms in both groups
Tamsha & Singh (2023)	India	*N* = 33	13–16	5 consecutive days, 45 minutes; no description of MI programme	Pre-post	No	No	Connors ADHD rating scale	Peer relations improvements

AAQ: Acceptance and Action Questionnaire; CAMM: Child and Adolescent Mindfulness Measure; CBCL: Child Behavior Checklist; CPT: Continuous Performance Test; DERS: Difficulties in Emotion Regulation Scale; FAD: Family Assessment Device; IM-P: Interpersonal Mindfulness in Parenting Scale; MAAS: Mindfulness Attention Awareness Scale; MBSR: Mindfulness Based Stress Reduction; MDI-C: Multi-score Depression Inventory for children; MI: mindfulness intervention; RCADS: Revised Child Anxiety and Depression Scale; SIPA: Stress Index for Parents of Adolescents; SNAP-IV: Swanson, Nolan and Pelham-IV; STAU: Treatment-as-usual; WASI: Wechsler Abbreviated Scale of Intelligence.

## Results

**Figure 1.**
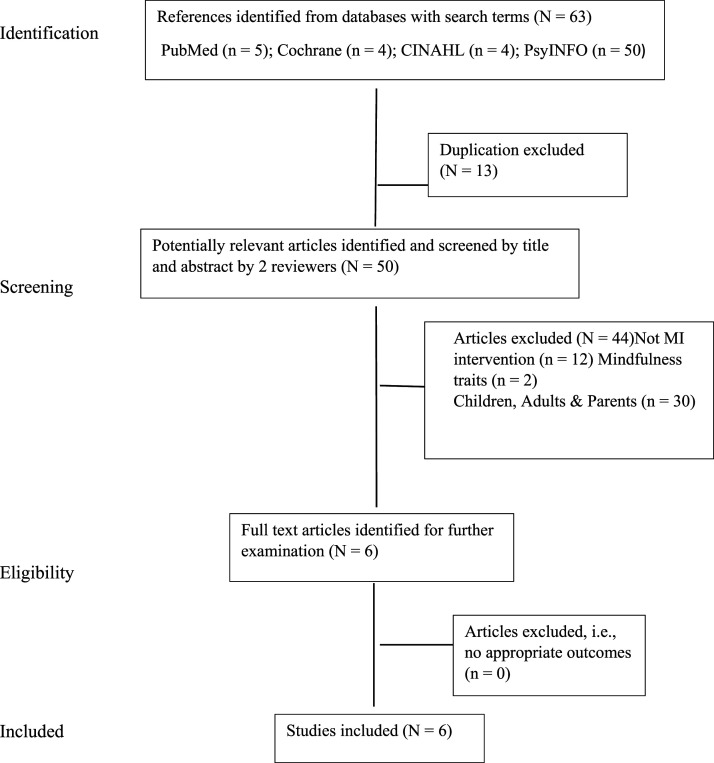
Flow diagram of the literature search.

The PRISMA flowchart describing the selection process, including reasons for exclusion is presented in Figure 1. The search retrieved a total of 63 articles, next, 13 duplicates were removed. Of the remaining articles (*N* = 50), based on titles/abstracts these were assessed for inclusion eligibility, whereby 30 studies were excluded due to different sample population (i.e., children under 12 years old, college/university students above 19 years old, parents only); and another 12 studies were excluded as these did not utilise mindfulness as the primary intervention (i.e., studies predominantly examined Cognitive Behavioural Therapy, Dialectical Behavioural Therapy, social skills training, Ritalin and family therapy). A further 2 studies were excluded as it examined dispositional/trait mindfulness via survey with no intervention, leaving 6 articles available for review (see Figure 1). The references of the 6 articles were screened and no further studies resulted. Study authors ranged from several countries including Iran (Abdolahzadeh et al., 2017; Kiani et al., 2017), Belgium (Roux et al., 2021; Roux & Philippot (2020), Canada (Hickdicky et al., 2015) and India (Tamsha & Singh, 2023); see Table 1 for a summary of the studies reviewed.

### Participants

All studies included adolescents (*N* = 290) with a diagnosis of ADHD, who have received mindfulness-based interventions; with one study included the parents (*N* = 17) who received in parallel mindfulness interventions. Studies were conducted in Belgium, Canada, Iran, and India; of which, two studies involved adolescents in residential services (Roux et al., 2021; Roux & Philippot, 2020); a further two studies involved female adolescents only (Abdolahzadeh et al., 2017; Kiani et al., 2017).

### Mindfulness-based interventions

From the studies, two types of mindfulness-based protocols were identified: (1) the adult versions (Mindfulness-Based Stress Reduction, MBSR – developed by Kabat-Zinn or Mindfulness-Based Cognitive Therapy, MBCT – developed by Segel, Williams & Teasdale, 2002) which include elements of mindful meditation practices incorporating mind-body awareness concepts, and (2) a modified adolescent MYmind group MBI version.

Four clinical studies utilised the adult MBSR or MBCT or a modified version, MYmind (Abdolahzadeh et al., 2017; Haydicky et al., 2015; Kiani et al., 2017; Tamsha & Singh, 2023). None of these studies reported day-long retreat (day-long retreat is one of the key features in adult mindfulness-based programmes). Of these, Haydicky et al. (2015) provided a detailed account of the modifications of the adopted adult mindfulness intervention/protocol, MYmind, which is based on adult MBCT (Segal, Williams & Teasdale, 2002). MYmind has been empirically validated, originally developed, and piloted in Netherlands (Bogels et al., 2008).

A further two studies by Belgium researchers (Roux et al., 2021; Roux & Philippot, 2020) examined MBI effectiveness on residential Adolescents with ADHD (*n* = 129 and *n* = 49), with a modified adolescent MBI. Their programme was drawn from two other adolescent group MBI programmes for enhancing adolescents’ emotional regulation (Deplus & Lahaye, 2015*; Taming the Adolescent Mind* programme, TAM; Tan & Martin, 2012). This programme consisted of two parts; part 1 included 6 sessions of emotion psychoeducation (introduction to emotion, calm the mind and body awareness); part 2 involved 10 sessions of mindfulness meditation. Their outline of the programme was comprehensively described in each of the studies.

In five studies, the length of the group MBI programmes ranged from 8 weeks to 10 weeks, with each session typically lasting for 90 minutes; with one exception where mindfulness sessions were delivered in block series, over 5 consecutive days and 45-minutes per session (Tamsha & Singh, 2023).

### Control

Four of the reviewed studies randomized participants to an intervention or control group, although no information regarding the randomization procedures were detailed (Abdolahzadeh et al., 2017; Kiani et al., 2017; Roux et al., 2021; Roux & Philippot, 2020). Four studies used a waitlist control group (Abdolahzadeh et al., 2017; Haydicky et al., 2015; Kiani et al., 2017; Roux & Philippot, 2020). Tamsha and Singh’s (2023) study had no control group. Only one study compared the effectiveness of MBI to a treatment-as-usual as control group (Roux et al., 2021), however no details of what treatment-as-usual entailed.

### Outcomes

Abdolahzadeh et al. (2017) included randomization of female participants (*N* = 30) into MBI treatment and waitlist control groups. At post-intervention, adolescents in the experimental condition reported improvements of ADHD symptoms as measured on the Swanson, Nolan and Pelham (SNAP) rating scale and an increase of mindfulness, as measured by the Mindful Attention Awareness Scale (MAAS) compared to adolescents in control condition (*p* = 0.001, F1,23 = 72.04). However, there was no follow-up, although gains were achieved for participants in the treatment condition (*n* = 15); two participants dropped out, leaving a small sample size and results limited to only female adolescents, constraining the generalisability of this study.

Haydicky et al. (2015) examined the effects of MYmind, a modified adult mindfulness training on adolescents with ADHD (*n* = 18) in an international school setting and their parents (*n* = 17) who also attended an 8-week parallel group session. This longitudinal study had four time points: baseline (i.e., 4 weeks before the intervention), pre- and post-intervention and at 6-week follow-up. After intervention, parents reported reductions in both their adolescents’ inattention conduct and peer relations problems, as measured on the Connors ADHD Rating Scale. The parents continued to report improvements in adolescent symptomatology; mindful parenting and parenting stress were also maintained at 6-week follow-up. The adolescents reported no improvements on any of the outcome variables during intervention period, but reductions in internalising problems were achieved at follow-up. Detailed analyses of the impact of mindfulness on parents were offered. However, like other studies it suffered from a small sample size and lacked randomised control group, making it difficult to assess the representativeness and generalisability of results. Furthermore, no mindfulness measures were obtained for the adolescents.

Kiani et al. (2017) evaluated the impact of MBI on executive functioning and emotion dysregulation in a 2 × 2 design with 30 Iranian (13 – 15-year-old) females only and excluded participants who were on medication. Participants were randomized into two groups – MBI v Control wait-list conditions. An 8-step Mindfulness for adult ADHD program was implemented in this study. The sessions consisted of mindfulness meditation practices, with themes on: Attention and the five senses: mindful breathing; mindfulness of sound, breath and body; Mindfulness of Thoughts; Mindfulness of Feelings, Listening and Speaking. Post tests results showed participants in treatment condition performed higher on inhibition, planning performance on executive functioning in laboratory tasks compared to control group. On emotion dysregulation, participants reported improvement at post treatment compared to participants in control condition (*F* (1, 27) = 6.41, *p* = .02).

Roux et al. (2021) explored MBI impact on both internalizing (anxiety and depression) and externalizing (inattention, hyperactivity and oppositional) symptoms of adolescents with ADHD in residential services (*N* = 129). Participants were allocated to one of the three conditions – treatment-as-usual (TAU), health psychoeducation (HP) or MBI condition; and data were compared over three time points, plus 1-year follow-up on self-report questionnaires and behavioural tasks. There were no differences on behavioural tasks between TAU, the MBI and the HP groups at post-intervention. This study included an age-appropriate mindfulness measure, CAMM (Child and Adolescent Mindfulness Measure); and the analyses revealed a main effect of time for the CAMM questionnaire, suggesting an increase in the mindfulness score between time 2 (midpoint of intervention, i.e., session 6) and time 3 (at post-intervention) for the three groups (*F* (1,120) = 8.36, *p* = 0.005). Also, a main effect of medication was found indicating that the mindfulness score was higher among participants who did not take medication.

Roux and Philippot (2020) examined MBI effects on ADHD impulsivity and depressive symptoms as well as ADHD in adolescents randomized into two conditions - MBI plus treatment-as-usual (MBI + TAU) versus waitlist control group of adolescents (TAU). Treatment-as-usual consisted of psychotherapy, speech therapy and physiotherapy; and did not exceed 3 hours per week for each participant. They recruited adolescents (*N* = 56) in a residential institution. Results showed that both groups decreased in depressive symptomatology as measured by Multiscore Depression Inventory for Children (MDI-C, Berndt and Kaiser, 1996) and participants in MBI + TAU condition decreased in impulsivity (*t* (21) = −2.03, *p* = 0.055) symptomatology.

Tamsha and Singh (2023) examined group MBI impact on peer relationships among adolescents with ADHD (*N* = 33) in a school setting. The pre-post experimental design, single condition design; the group MBI treatment included 45-minutes mindfulness practice each session for five days in a week. They found significant progress from pre-to post intervention with a mean difference of 5.51 (*t* (32) = 21.38, *p* < .001). There were no details of the group MBI programme, mindfulness measures, or control condition in this study.

## Discussions

Given the plethora of mindfulness publications, it was appropriate to examine whether this form of training is effective in improving ADHD. This scoping review examined clinical evidence on the effectiveness of group MBI in improving attention in adolescents with ADHD. We reviewed six studies, of which four were two-group wait-list control design, one study was a two-group wait-list quasi experimental design and the remaining study a single group design. In general, adolescents with ADHD randomly assigned to a group MBI condition showed mixed results in decrease in ADHD symptoms in inattention and impulsive behaviours relative to in two-group wait-list adolescents with ADHD in the control groups.

Our results are consistent with the reviews found in previous studies on children with ADHD where substantial methodological shortcomings and variability in design and programme content were evidenced (Bogels et al., 2008). Overall, It is unclear whether the studies examined were adequately powered, i.e., small sample size (with exception of Roux et al., 2021); three studies included only females or residential male participants; and one study (Tamsha & Singh, 2023) had no control group making it difficult to assess the representativeness and generalisability of results.

Other methodological shortcomings include outcome measurements. Only one study administered an age-appropriate mindfulness measure (Roux et al., 2021), whilst the majority either omitted entirely (Haydicky et al., 2015; Kiani et al., 2017; Tamsha & Singh, 2023) or used adult mindfulness scale, for example MAAS (Abdolahzadeh et al., 2017).

Parallel group mindfulness-based parenting intervention demonstrates clinical utility and further directions are needed to shed light on the effects of group MBI for adolescent ADHD; even when the core ADHD outcomes in some studies were not significantly reduced in the adolescents (Haydicky et al., 2015). It is possible that a decrease in parental stress may facilitate different interaction between parents and their adolescents. For example, behaviours that would have been irritating, are observed from a different perspective and their adolescent’s emotional needs/state observed more objectively. Previous studies where mindfulness family-based interventions were administered to both parents and younger children concurrently, suggested an improvement of non-escalation of potentially negative interactions with their children (Bogels, et al., 2014). Currently, few studies incorporate co-joint adolescent-parent mindfulness training and more confirmatory data is required.

Additionally, the plethora of published mindfulness meditation studies have focused on recent standardized MBIs, such as MBCT and MBSR developed for adult population, compared to a handful of programmes that have been developed or modified for adolescents (Burke, 2010; Tan 2015). It is worth mentioning that for centuries meditation has been primarily taught within context of traditional spiritual paths and/or in intensive retreats e.g., Vipassana and Shamatha practices (Rapgay, 2009); and although day-long retreats have been incorporated in adult MBI programmes, this feature has not been adopted in the studies reviewed here.

Although most studies here supported group MBI’s value for adolescents with ADHD, none of the studies measured participants’ adherence of mindfulness practice or out-of-session mindfulness practices. According to Voils et al. (2012), *“Dosing is potentially the most important decision that must be made when building or refining behavioural interventions”* (p. 1225) and it should be operationally defined by duration, frequency, and amount. Before the Voils et al. (2012) study, there was no standardization of dosing terminology or reporting in social science behavioural interventions. Clinical research in mindfulness has mixed reports of intervention details in terms of duration, frequency, and amount, making it difficult to replicate studies and to determine the minimum amount of mindfulness training necessary to affect the positive changes often reported (Bambacus & Conley, 2021). Dosage of mindfulness training is not well measured and rarely discussed in the literature; understanding the optimal training dosage for improving attention would extend MBI effectiveness.

The effect of group MBI on symptoms of ADHD may be influenced by other comorbid affective disorders (depression and anxiety) and ADHD subtypes. Overall, studies reviewed included generic, and broad-based statistical reporting of global ADHD symptoms. Future directions may examine specificity, including subgroup analyses focussing on the components of MBI that work effectively in the treatment of ADHD - for example which mindfulness meditation technique or combination thereof is more effective targeting specific core symptoms of ADHD (i.e., hyperactivity, impulsivity, inattention). Currently, there are few component analyses in mindfulness meditation field that examines interaction between meditation type (Fincham et al., 2023). It is possible different mindfulness meditation techniques may impact differently on ADHD subtypes. Dismantling studies examining the effects of different mindfulness techniques (i.e., body scan, mindful movements, mindful breathing) and how these may work on different psychological processes would yield intriguing insights.

These concerns are important before we can draw any conclusions on the efficacy and effectiveness of group MBI for adolescents with ADHD. Apart from dosage of mindfulness practice, other limitations include a lack of clarity around participants’ ADHD clinical presentations and/or co-morbidities and inconsistent reporting of medication status. The clinical heterogeneity of ADHD and the difficulty this presents in research maybe overcome with recent clinical guidelines specific to ADHD (Coghill et al., 2023; May et al., 2023). Such guidelines could assist in promoting development and evaluation of clinical recommendations and inform future research.

Beyond attention training in adolescents with ADHD, self-directedness has an important place in psychological health, demonstrating a strong association of positive mental health (Cloninger, 2006). It can be argued that self-directedness may play an important role in adolescent’s development and has its place in mindfulness training. Future studies examining the relationship between mindfulness training on self-directedness and ADHD may be illuminating.

Finally, mindfulness interventions in ADHD treatment have predominantly centred on therapeutic effect and less on exploring the underlying mechanisms. With advancements in methodologies, e.g., neuroimaging-based, and computational modelling analyses, it is timely to explore and generate understanding of the psychological facets of mindfulness, such as acceptance, awareness, and mindfulness.

## Conclusions

This review examined effectiveness of group based MBI for adolescents with ADHD. Although studies here added value in our understanding, caution about group based MBI effectiveness is warranted due to the low methodological quality of the studies reviewed. Future studies should consider improvement in study design, such as larger scale multisite studies and trials, and treatment specificity. Nonetheless, the continued interest provides important contribution to building an evidence base and the findings in this review provides a panorama of MBI for adolescents with ADHD.
